# Preparation and Characterization of a Novel Polysaccharide-Iron(III) Complex in* Auricularia auricula* Potentially Used as an Iron Supplement

**DOI:** 10.1155/2019/6416941

**Published:** 2019-06-11

**Authors:** Tong Liu, Tingting Liu, Hongcheng Liu, Hongxiu Fan, Bingyu Chen, Dawei Wang, Yanrong Zhang, Fengjie Sun

**Affiliations:** ^1^School of Food Science and Engineering, Jilin Agricultural University, Changchun, China; ^2^School of Science and Technology, Georgia Gwinnett College, Lawrenceville, GA, USA

## Abstract

Iron deficiency anemia has been a widespread disease. As an effective and stable iron supplement, the physiochemical properties of the polysaccharide iron complex have been widely studied. In this study, we characterized a novel polysaccharide-iron(III) complex extracted in an edible fungal species* Auricularia auricular* (AAPS-iron(III)). The highest iron content (28.40%) in the AAPS-iron(III) complex was obtained under the optimized preparation conditions including an AAPS to FeCl_3*∙*_6H_2_O ratio of 2:3 (w/w), a pH value of 8.0 in solution, a reaction temperature of 50°C, and a reaction time of 3 h. The physical and chemical properties of the AAPS-iron(III) complex were characterized by qualitative and quantitative analyses using scanning electron microscope, particle size distribution, thermogravimetric analyzer, Fourier transform infrared spectroscopy, circular dichroism, and ^1^H nuclear magnetic resonance. Result showed that, although the iron was bound to the polysaccharide, it was released under artificial gastrointestinal conditions. The AAPS-iron(III) complex exhibited high stability (under 50-256°C) and water solubility. The AAPS-iron(III) complex also showed high antioxidant activity in vitro, demonstrating an additional health benefit over other typical nonantioxidant iron nutritional supplements. Furthermore, the AAPS-iron(III) complex showed high efficiency on the treatment of the iron deficiency anemia in the model rats. Therefore, the AAPS-iron(III) complex can be used as a nutritional fortifier to supply iron in industrial processing and to assist the treatment of iron deficiency anemia.

## 1. Introduction

Iron is one of the most important minerals in the human body. As an auxiliary factor of metabolisms and functions of the immune system, iron plays an important role in maintaining the normal functions of organisms [1]. The World Health Organization has reported that 46% of children of 5-14 years old and 48% of pregnant women worldwide have symptoms of anemia, most of which are caused by iron deficiency [2]. The main causes of iron deficiency anemia include insufficient intake of iron from the diet, poor absorption of iron, increased physiological demand for iron, and excessive loss of iron. To compensate for the iron deficiency, ferrous salts such as ferrous sulfate, iron fumarate, and ferrous gluconate are commonly used as oral iron supplements [3, 4]. However, long-term use of oral iron supplements may cause gastrointestinal side effects [5] and iron-induced oxidative stress [6]. Studies have also shown that the gastrointestinal side effects caused by the long-term use of therapeutic ferrous sulfate oral agents may include nausea, vomiting, abdominal pain, and constipation [7–10]. Therefore, it is imperative to develop new iron supplements without the side effects.

The interest of polysaccharides of natural origin has expanded into the areas of anticancer, antitumor, antioxidant, antiobesity, neuroprotective, antimicrobial, and anti-inflammatory, with broadened fields of applications of these polysaccharides in food supplements, cosmetics, pharmaceuticals, and biomedical uses [11, 12]. Recent studies have documented polysaccharide-iron(III) complexes with high stability and increased bioavailability in comparison to those of currently commonly used oral iron supplement, e.g., ferrous sulfate [13]. Polysaccharide-iron complexes have been the most studied among all of the iron(III) complexes because they have high stability and water solubility, and more important, reduced side effects [14, 15]. Polysaccharide-iron complexes have also been used as iron supplements for treatment of iron deficiency anemia because of their relatively high bioavailability and significant reduction of side effects, e.g., gastrointestinal discomfort and oxidative stress induced by a high concentration of iron(III) absorption, in comparison with those of traditional oral iron supplements [16].

*Auricularia auricula* is a type of black saprophytic fungus that grows on decayed wood. This species is also commonly known as “wood ear” or “ear fungus” in Chinese, which literally means “the ear of the wood,” due to the shape of its body. As a medicinal and edible fungus,* Auricularia auricula* has been produced mainly in China but widely consumed for over 1000 years worldwide. Although the species* Auricularia auricula *is widely planted in China [17], a large amount of the products is wasted due to the oversupply on the market, making it easier and cheaper to obtain this species of fungus in comparison to other sources of complexing agents. The most abundant functional components in this species of fungus are* Auricularia auricula* polysaccharides, which have been served as antioxidants [18–20], anticoagulants [21, 22], antitumor agents [23, 24], and blood lipid reducers [25]. In our current study,* Auricularia auricula* polysaccharides have shown high levels of bioactivity and could be potentially used as ligands for iron(III) to promote its absorption in the digestive system, significantly increasing the value of* Auricularia auricula* in both industrial and medicinal areas.

In this study, we have prepared and characterized the physicochemical properties of a novel polysaccharide iron(III) complex in* Auricularia auricular* (AAPS-iron(III)) using scanning electron microscope (SEM), thermogravimetric analyzer, Fourier transform infrared spectroscopy (FTIR), circular dichroism (CD), and ^1^H nuclear magnetic resonance (^1^H NMR). The bioavailability and antioxidant activity of the AAPS-iron(III) complex were studied by various in vitro assays. We have also focused on optimizing the preparation conditions of this complex.

## 2. Materials and Methods

### 2.1. Materials and Chemicals

Samples of* Auricularia auricula* were obtained from The Engineering Research Centre of Chinese Ministry of Education for Edible and Medicinal Fungi (Changchun, China). 1,1-diphenyl-2-picrylhydrazyl (DPPH) and 2,2'-azino-bis-(3-ethylbenzthiazoline-6-sulfonic) (ABTS) were purchased from Sigma Chemical Co. (St. Louis, MO, USA). All other analytical grade chemical reagents were purchased from the Beijing Chemical Reagent Factory, China.

### 2.2. Purification of the* Auricularia auricula* Polysaccharide (AAPS)

Polysaccharides of* Auricularia auricula* were obtained from the fungus using ultrasonic microwave (240W) together with hot water (85°C) extraction, protein and pigment removal with macroporous resin method, and alcohol (80% v/v) precipitation. The extraction yield was 21.25% and the polysaccharide content of the sample was 70.58%. As the main fractions, AAPS was isolated from the sample by diethylaminoethyl (DEAE) cellulose-52 chromatography and Sephadex G-100 gel filtration chromatography. The final yield of AAPS was 42.86% with the polysaccharide content of 95.35%. The monosaccharide composition of the AAPS was determined by the precolumn derivatization of High Performance Liquid Chromatography (HPLC) as an acidic heteropolysaccharide, mainly composed of mannose, glucuronic acid, glucose, and xylose with the molar ratio of 3.62:7.23:12.57:4.24 and the relative molecular weight of 3.783 × 105 Da.

### 2.3. Preparation of the AAPS-Iron(III) Complex

The AAPS-iron(III) complex was prepared and characterized according to a modified method of Dong et al. [26]. The AAPS-iron(III) complex was formed by neutralization of an FeCl_3_ carbohydrate solution. Briefly, 0.3 mL of FeCl_3_·6H_2_O (2 M) was added dropwise with continuous stirring to an aqueous solution containing 2% (w:w) of AAPS and 1.5% (w:w) of sodium citrate. The pH was adjusted to 6.0, 7.0, 8.0, 9.0, and 10.0, respectively, by adding either HCl (2 M) or NaOH (2 M). The temperature was set at 30°C, 40°C, 50°C, 60°C, and 70°C, respectively, using a constant temperature oscillation incubator. After 3, 4, 5, 6, 7, 8, and 9 h, respectively, the reaction mixture was centrifuged at 4000×* g* for 10 min. The supernatant was concentrated and subsequently dialyzed in distilled water to remove the unbound ions. The retentate was condensed and the solid portions were precipitated using anhydrous ethanol and then freeze-dried for further experiments.

### 2.4. Qualitative Identification of AAPS-Iron(III) Complex

Ten milligrams of dried AAPS-iron(III) complex was dispersed in 2 mL of H_2_O and the resulting mixture was stirred at room temperature for 2 h. A few drops of potassium thiocyanate were then added to the solution and the reaction phenomenon was observed and recorded. In a similar manner, 10 mg of dried AAPS-iron(III) complex was dispersed in 2 mL of HCl (1 M) and stirred at room temperature for 2 h. A few drops of potassium ferrocyanide were then added and the reaction phenomenon was observed and recorded.

### 2.5. Quantification of Iron Content

Quantification of the iron content was performed according to the method of Pitarresi et al. [27] with minor modifications. Ten milligrams of dried AAPS or AAPS-iron(III) complex was dispersed in 20 mL of HCl (1 M) by stirring for 24 h in order to break the compounds to release the iron. Then, mix 1 mL of each solution with 1 mL of hydroxylamine hydrochloride (10%), 2.5 mL 1,10-phenanthroline (10%), and 5 mL sodium acetate trihydrate buffer solution (pH 4.5). The mixed solution was subjected to UV–vis analysis (UV-2450 spectrophotometer, Shimadzu, Japan). The iron content in the AAPS-iron(III) complex was calculated from the absorbance at 510 nm. The calibration curve was obtained by using the standard solutions of ferrous ammonium sulfate in distilled water based on the following formulate: y = 0.0045x – 0.170, where y is the absorbance at 510 nm and x is the content of iron (R^2^ = 0.9995).

### 2.6. Physicochemical Characterization of the AAPS-Iron(III) Complex

#### 2.6.1. SEM Analysis

The powdered AAPS or AAPS-iron(III) complex was observed using an environmental scanning electron microscope (SSX-550, Shimadzu, Japan). Samples were evenly dispersed on a sample stage covered with a double-sided adhesive and sprayed with gold powder. The accelerated voltage was set to 5.0 kV and each sample was observed at 500× and 20,000× magnifications.

#### 2.6.2. Particle Size Distribution and Molecular Weight Analysis

AAPS and the AAPS-iron(III) complex were each dispersed in ultrapure water to make the solutions (2% g/ml). The particle size distributions of the samples were determined by a laser particle size analyzer (Mastersizer3000, Malvern Instruments Ltd, United Kingdom) with a shading rate of 10-20% and scattering intensity of 35. The relative molecular weights of AAPS and AAPS-iron(III) complex were estimated on an Agilent 1100 HPLC system equipped with a refractive index detector (RID) and a PL aquagel-OH Column (79911GF-083, Agilent). The sample was filtered on a 0.45-*μ*m pore membrane before injection (20 *μ*L) and eluted with Na_2_SO_4_ solution (0.1 M) in PBS buffer (0.01 M, pH 6.8) at a flow rate of 0.7 mL/min. The column temperature was maintained at 40°C.

#### 2.6.3. Thermogravimetric Analysis (TGA)

The desired sample (5 mg) was placed on an aluminum sample plate of the thermogravimetric analyzer (TGA4000, PerkinElmer, USA). Nitrogen (99.99%) with a flow rate of 30 mL/min was used as a carrier gas. The detection temperature was raised from 50°C to 650°C at a heating rate of 10°C/min.

### 2.7. Spectroscopic Characterization of the AAPS-Iron(III) Complex

#### 2.7.1. Fourier Transform Infrared Spectroscopy (FTIR) Analysis

Infrared spectral analysis was performed using an FTIR spectrometer (IR Prestige-21, Shimadzu, Japan). Two milligrams of AAPS or the AAPS-iron(III) complex was dried with 200 mg potassium bromide at 105°C, mixed uniformly, and compacted into a disk for the collection of infrared spectra at 4000–400 cm^−1^. The spectra were recorded with a resolution of 4 cm^−1^ and a coaddition of 16 scans and were analyzed using IRSolution (Shimadzu Optics Limited, Japan).

#### 2.7.2. Circular Dichroism (CD) Analysis

The CD spectra of AAPS and the AAPS-iron(III) complex solutions (1.0 mg/mL) were measured on a J-820CD spectropolarimeter (JASCO, Japan). Each CD spectrum represented the accumulation of three scans at 100 nm/min with a time constant of 1 s and a slit width of 1 nm. Data were collected from 185 to 300 nm with an interval of 1 nm.

#### 2.7.3. ^1^H Nuclear Magnetic (^1^H NMR) Analysis

The ^1^H NMR spectra of AAPS and the AAPS-iron(III) complex were recorded in pure D_2_O (Cambridge Isotope Laboratory, Inc., USA) and at room temperature using a Bruker Advance 600 MHz NMR spectrometer (Bruker, Germany). 500 and 1000 scans were used for AAPS and the AAPS-iron(III) complex, respectively.

### 2.8. Iron Release In Vitro Assay of the AAPS-Iron(III) Complex

Artificial gastric juice was prepared according to Hasan et al. [28]. Sodium chloride (2.0 g) and pepsin (3.2 g) were dissolved in quadruple-distilled water. After adding 80 mL of 1 M HCl, additional quadruple-distilled water was added to obtain the final volume of 1000 mL (pH 2.0). Artificial intestinal juice (pH 8.0) was prepared by dissolving 5.0 mL each of bile salt mixture (25.0 g/L) and pancreatic extract (4.0 g/L) in NaHCO_3_ (1 M), followed by adding quadruple-distilled water to reach the final volume of 1000 mL [29]. Iron release from the AAPS-iron(III) complex was measured according to the modified method of AlKhatib et al. [30]. Two types of dissolution media were used during the two successive stages of iron release. First, 50 mg of the AAPS-iron(III) complex was pulverized and placed in a 500-ml flask for 2 h with the artificial gastric juice (pH 2.0). Then, the solids were removed from the artificial gastric juice and, without cleaning, placed in a flask containing 500 mL of the artificial intestinal juice (pH 8.0) for 3 h. The dissolution media were maintained at 37°C throughout the process. Samples (10 mL) were withdrawn at predetermined time intervals for analysis of the iron ions released. The withdrawn volumes were immediately replaced by the equivalent volumes of fresh medium to maintain the overall volume. The sample solution was filtered through a 0.45-*μ*m pore membrane and analyzed using the method of Pitarresi et al. [27] on 2,5-phenanthroline to calculate the amount of iron released.

### 2.9. Antioxidant Activities of the AAPS-Iron(III) Complex

#### 2.9.1. DPPH Radical Scavenging Activity

The DPPH radical scavenging activity was determined according to the methods of Chen et al. [31] and Zhang et al. [32] with minor modifications. A DPPH solution (2 mmol/L) was prepared in anhydrous ethanol in advance. A sample (1.0 mL) of the AAPS-iron(III) complex in distilled water (at a series of concentrations of 0.0, 0.1, 0.2, 0.3, 0.4, 0.5, 0.6, 0.7, 0.8, 0.9, and 1.0 mg/mL) was added to 1.6 mL of distilled water and 0.4 mL of DPPH solution (2 mM). The mixture was shaken in a water bath (30°C) under dim light and incubated for 10 min. Deionized water was used as the blank control sample and ascorbic acid as the positive control sample. The absorbance of the reaction system was measured at 517 nm. The DPPH radical scavenging activity was assessed using the following equation: DPPH radical scavenging ability (%) = [1–(A_1_–A_2_)/A_0_]*∗*100, where A_1_ is the absorbance of the sample or ascorbic acid with the DPPH solution, A_2_ is the absorbance of the mixture with deionized water, and A_0_ is the absorbance of the blank control.

#### 2.9.2. ABTS Radical Scavenging Activity

The ABTS radical scavenging activity was evaluated according to the method of Liang et al. [33] with some modifications. ABTS (0.3841 g) and K_2_S_2_O_8_ (0.0662 g) were dissolved in deionized water and the resulting solution was transferred to a 100-mL volumetric flask and diluted with deionized water to a final volume of 100 mL. The mixture was then incubated in dark for 12 to 16 h at room temperature. A stock solution of ABTS (7.0 mmol/L) and 2.45 K_2_S_2_O_8_ (2.45 mmol/L) was diluted with PBS (0.01 mol/L, pH 7.4) to a concentration that provided an absorbance of 0.7 at 734 nm. In the reaction system, 1.0 mL of H_2_O and 2.0 mL of the ABTS solution were added to 1.0 mL of the sample solution of AAPS, AAPS-iron(III) complex, or ascorbic acid in distilled water to make a series of concentrations of 0, 0.1, 0.2, 0.3, 0.4, 0.5, 0.6, 0.7, 0.8, 0.9, and 1.0 mg/mL, respectively. Each solution was shaken well and placed in dark for 6 min at room temperature, and then the absorbance of the mixture was measured at 734 nm. Deionized water was used as the blank control and ascorbic acid as the positive control. The ABTS radical scavenging activity was calculated based on the following equation: ABTS radical scavenging ability (%) = [1–(A_1_–A_2_)/A_0_]*∗*100, where A_1_ is the absorbance of the sample solution or ascorbic acid with ABTS, A_2_ is the absorbance of the sample solution or ascorbic acid with deionized water, and A_0_ is the absorbance of the blank control.

#### 2.9.3. Inhibition Assay of Liver Lipid Peroxidation

Inhibition of lipid peroxidation in livers of Kunming rats (weighed 18-20 g, provided by the Changchun Institute of Biological Products Co., Ltd., Changchun, China) was evaluated according to the method of Ma et al. [34] with some modifications. Rats were sacrificed and the livers were rapidly washed and homogenized at 4°C in a volume of normal saline that was 25 times the amount of the volume of the livers. One milliliter of sample solution of AAPS, AAPS-iron(III) complex, or ascorbic acid (with a series of concentrations of 0, 0.1, 0.2, 0.3, 0.4, 0.5, 0.6, 0.7, 0.8, 0.9, and 1.0 mg/mL in distilled water) was mixed separately with 1.0 mL of mouse liver homogenate, 0.1 mL of FeSO_4_ (6 mM), and 0.05 mL of H_2_O_2_ (60 mM), and the mixtures were incubated at 37°C for 1.5 h. Each mixture was added to 1.0 mL of trichloroacetic acid solution (10%, w/v) and 1.0 mL thiobarbituric acid solution (0.67%, w/v), mixed and boiled for 15 min, and then cooled with water. After centrifugation at 4000 rpm for 5 min, the absorbance of the supernatant was measured at 532 nm using deionized water as a blank control. Malondialdehyde (MDA) is an aldehyde-based by-product produced by lipid peroxidation which causes strong chemical toxicity to cells. Therefore, MDA production is used as an indicator of the level of lipid peroxidation [35]. The inhibition ratio of MDA was calculated according to the following equation: inhibition ratio of MDA (%) = [1–(A_1_–A_2_)/A_0_]*∗*100, where A_1_ is the absorbance of the mixture with the sample solution or ascorbic acid, A_2_ is the absorbance of the sample solution with deionized water, and A_0_ is the absorbance of the blank control.

### 2.10. Antianemia Function Test of the AAPS-Iron(III) Complex

The rat model of iron deficiency anemia was established according to Yun et al. [36] with minor modifications. Sixty Wistar rats (30 males and 30 females, 6-8 weeks old, weighed 200±20 g, provided by the Changchun Institute of Biological Products Co., Ltd., Changchun, China) were fed adaptively for three days prior to the measurements of the blood parameters using a Hitachi 7080 automatic biochemical analyzer (Hitachi, Japan). The number of red blood cells (RBC), hemoglobin (HB), mean corpuscular volume (MCV), mean hemoglobin content (MCH), and red blood cell volume distribution width (RDW) were measured as normal values prior to the model establishment. The 60 rats were randomly divided into two groups: 10 rats were fed with basic diet as blank control (BC) group and the other 50 rats (the treatment group) were fed with low iron diet for four weeks and blood samples were collected from tail vein twice a week for 8 times. Both groups were fed with free water. Stainless steel cages are used to keep the rats in order to prevent iron pollution. In four weeks, RBC, HB, MCV, MCH, and RDW were measured by sampling the blood from the tail vein. A model rat is considered successful if the value of HB is less than 100 g/L.

#### 2.10.1. Animal Grouping and Treatment

The 60 model rats of iron deficiency anemia were randomly divided into five groups based on the amount of AAPS-iron(III) complex in their diet: model control group (MC), positive control group (PC), high dose group (HI), medium dose group (MI), and low dose group (LW). The PC group was fed with ferrous sulfate (8.3 mg/kg). The HI, MI, and LW groups were fed with AAPS-iron(III) complex of 16.4 mg/kg, 8.2 mg/kg, and 4.1 mg/kg, respectively. Except for the BC group, other five groups continued to be fed with low iron diet. All animals were given the tested drugs based on the intragastric volume of 10 ml/kg. The BC and MC groups were given distilled water of the same volume once a day for five weeks. Once the experiments started, the general observations of the animals were made. The body weight of the animals was measured every 7 days and the state of the animals was observed. Five weeks after administration, abdominal aorta blood samples were collected at 3500 r/min to determine the contents of erythrocyte free protoporphyrin (FP), HB, RBC, Hematocrit (HCT), MCV, MCH, and the mean corpuscular hemoglobin concentration (MCHC) in erythrocyte.

### 2.11. Statistical Analysis

All experiments were repeated three times. The results were shown as average ± standard deviation. SPSS (version 18.0, IBM Corp., Armonk, NY, USA) was used to perform the one-way analysis of variance (ANOVA) and the least significance difference (LSD) Duncan analysis. A statistically significant difference is indicated by p<0.05 or p<0.01.

## 3. Results and Discussion

### 3.1. Physicochemical Characterization of the AAPS-Iron(III) Complex

#### 3.1.1. Preparation of the AAPS-Iron(III) Complex

Morphological observations showed that both AAPS and the AAPS-iron(III) complex were odorless, while AAPS was white granular power and the AAPS-iron(III) complex was reddish brown amorphous powder. The preparation of the AAPS-iron(III) complex has been previously reported in* Astragalus membranaceus* [37]. Under weak alkaline conditions, the iron ions polymerize through an oxygen bridge or a hydroxyl bridge. Citric acid is then released during the polymerization of the ferric citrate. Under alkaline conditions, polysaccharides interact with iron on the surface of the polymerized ferric citrate to form the polysaccharide-iron complex. In our study, results showed that, after adding ferric chloride to the solution of* Auricularia auricula* polysaccharides with sodium citrate, iron ions react with citrate ions to form a ferric citrate complex. The reaction system is kept in an alkaline environment through dropwise addition of sodium hydroxide solution throughout the experiments. The optimum preparation conditions of the AAPS-iron(III) complex included a ratio of AAPS to FeCl_3_*∙*6H_2_O of 2:3 (w/w), pH 8.0, reaction temperature of 50°C, and reaction time of 3 h. The AAPS-iron(III) complex had a relatively high iron content (28.40%) compared to those previously reported and showed increased water solubility. For example, Xu et al. [13] have obtained iron content of 24.15% in the AAPS-iron(III) complex in a fungal species of* Grifola frondosa* under the optimum preparation conditions based on the single factor and orthogonal optimization experiments, including a ratio of AAPS to the catalyst (sodium citrate) of 1:1 (g/g), reaction temperature of 80°C, reaction time of 1.5 h, and pH 8.0.

#### 3.1.2. Qualitative Identification of the AAPS-Iron(III) Complex

When drops of potassium ferrocyanide or potassium thiocyanate reagents were added to an aqueous solution of the AAPS-iron(III) complex, the appearance of the aqueous solution did not change (data not shown). In contrast, when potassium ferrocyanide or potassium thiocyanate was added to the AAPS-iron(III) complex in a hydrochloric acid solution, a dark blue precipitate and a blood red flocculent appeared, respectively (data not shown). These results clearly show that the AAPS-iron(III) complex does not contain free iron ions and the polysaccharide and iron were bonded rather than simply physically mixing together. The bond between the polysaccharide and iron was broken by strong acid to release the iron ions, resulting in the qualitative identification of the AAPS-iron(III) complex.

#### 3.1.3. SEM Analysis

**Figure 1:**
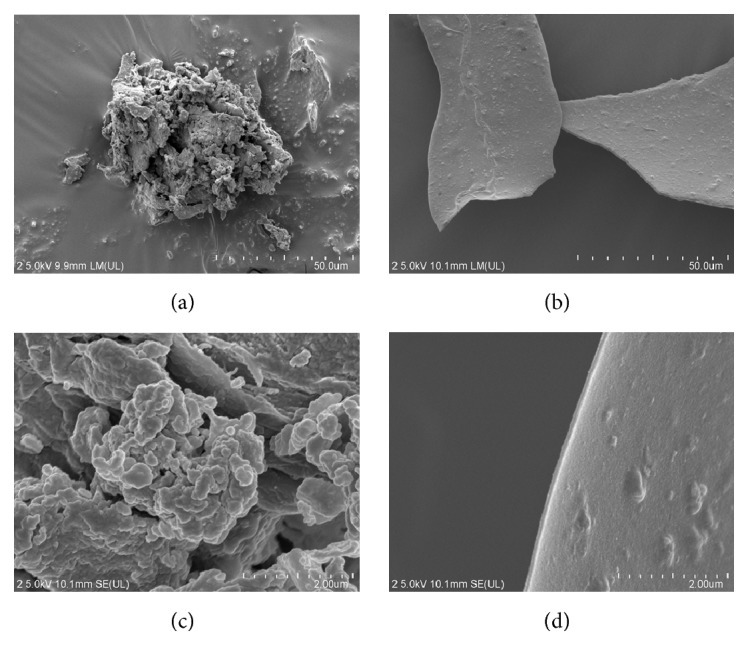
SEM images of AAPS ((a) and (c)) and the AAPS-iron(III) complex ((b) and (d)) with a sample thickness of about 0.1 *μ*m. 500× magnification in (a) and (b); 20,000× in (c) and (d).

Microphotographs showed that the morphologies of the fragmented AAPS and AAPS-iron(III) complex granules are very different (Figure 1). The polysaccharide of AAPS is flocculent with a rough surface (Figures 1(a) and 1(c)), while the AAPS-iron(III) complex shows a sheet-like structure with a smooth surface and neat edges (Figures 1(b) and 1(d)). These morphological observations may be due to the interactions of the polysaccharides with iron, changing the internal structure of the polysaccharides and subsequently their morphological appearances. Similar SEM observations have also been reported in the increased surface in the AAPS-iron(III) complex in* Astragalus membranaceus* [37].

#### 3.1.4. Particle Size Distribution and Molecular Weight Analysis

**Figure 2:**
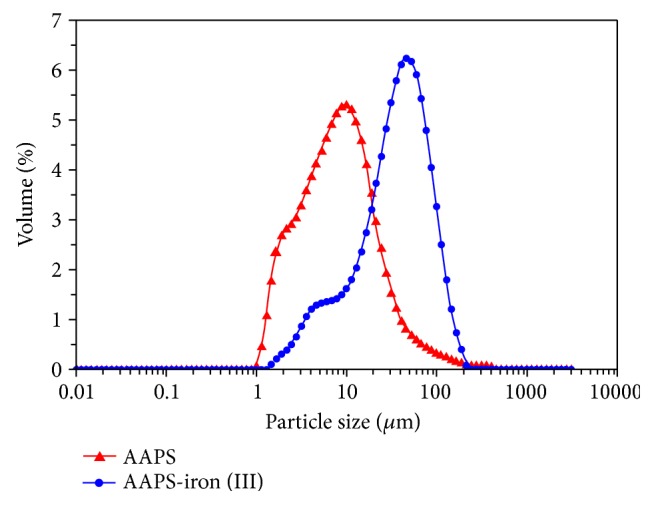
Particle size distribution of AAPS and the AAPS-iron(III) complex.

The particle size distribution index D_50_ values were 18 *μ*m and 96 *μ*m for AAPS and AAPS-iron(III), respectively (Figure 2). The average molecular weights of AAPS and AAPS-iron(III) complex were estimated to be 3.783 × 10^5^ Da and 8.708 × 10^5^ Da, respectively. The particle sizes and average molecular weights of the AAPS-iron(III) complex were significantly larger than those of AAPS (Figure 2), which was also observed in the SEM analysis (Figure 1). These results could be explained by the structural change of polysaccharide caused by its complexation with iron, resulting in significant differences in the particle size between AAPS and the AAPS-iron(III) complex. Comparable particle size distribution index D_50_ values of AAPS and AAPS-iron(III) complex (20 *μ*m and 104 *μ*m, respectively) were also reported in* Inonotus obliquus* [38].

#### 3.1.5. Thermogravimetric Analysis (TGA)

**Figure 3:**
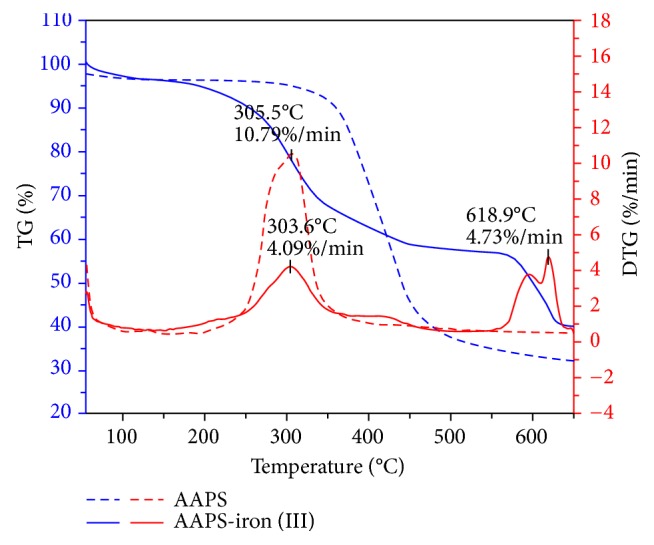
Thermogravimetry (TG) and derivative thermogravimetry (DTG) curves of AAPS and the AAPS-iron(III) complex.

The thermal stabilities of AAPS and the AAPS-iron(III) complex were investigated by thermogravimetric analysis (Figure 3). As previously reported [39], thermogravimetry (TG) curve was used to reflect the relationships between the sample weight and temperature, while the derivative thermogravimetry (DTG) curve was used to describe the relationships between the temperature and the rate of material weight changes upon heating. In our study, when temperatures increased from 50°C to 265°C, the mass loss of AAPS was primarily the free water and the rate of weight loss was 95.7%. AAPS decomposed drastically from 265 to ~500°C. For example, the rate of mass loss of AAPS reached 10.79% at 305.5°C. The rates of mass loss of AAPS decreased and tended to be stable at temperatures higher than 550°C. The final residual mass of AAPS was 28.8%. From 50°C to 256°C, the mass loss of the AAPS-iron(III) complex was the free water and the rate of weight loss was 94.6%, comparable to that of AAPS. The AAPS-iron(III) complex underwent the first rapid thermal decomposition from 256 to 500°C. At 303.6°C, the rate of mass loss was 4.09%/min and the rate of weight loss was 56.5%. A second thermal decomposition of the AAPS-iron(III) complex, stronger than the first one, occurred from 574 to 635°C. At 618.9°C, the rate of mass loss of the AAPS-iron(III) complex was 4.73%/min, whereupon the rate of mass loss of the AAPS-iron(III) complex tended to decrease until the final residual mass reached 38.9%. These results show that the residual mass of the AAPS-iron(III) complex was significantly higher than that of AAPS, likely due to the high content of iron in the AAPS-iron(III) complex. Therefore, it is reasonable to speculate that the complexation of AAPS with iron ions causes the two thermal decompositions of the APPS-iron(III) complex. These results demonstrate that the AAPS-iron(III) complex maintained high thermal stability in the temperature ranging from 50°C to 256°C, indicating that the AAPS-iron(III) complex is sufficiently stable if used as a type of nutritional fortifiers which are generally required to be stable below 250°C in the food industry. Similar thermal stability was also reported in* Grifola frondosa* [13], where the AAPS-iron(III) complex showed increased high stability in the temperature ranging from room temperature to 275°C in comparison to AAPS.

### 3.2. Spectroscopic Characterization of the AAPS-Iron(III) Complex

#### 3.2.1. FTIR Analysis

**Figure 4:**
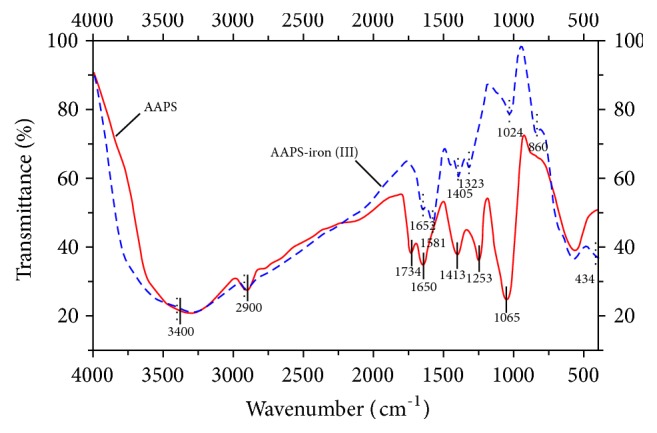
FTIR spectra of AAPS and the AAPS-iron(III) complex.

The FTIR spectra of AAPS and the AAPS-iron(III) complex (Figure 4) showed that iron complexing did not significantly disrupt the structures of the functional groups in AAPS of the AAPS-iron(III) complex. Similarly, the results by Wang et al. [40] also showed the congruence between the infrared spectra of both the polysaccharide and the polysaccharide-chromium(III) complex in* Inonotus obliquus*, indicating that the structure of the polysaccharide was not destroyed in the polysaccharide-chromium(III) complex. Based on these results, it is predicted that the structure (i.e., the functional groups) of the polysaccharide is not destroyed when complexing with the metal ion. The absorption peaks of AAPS and the AAPS-iron(III) complex at 3400 cm^−1^ were previously assigned to O-H stretching vibrations in* Ulva pertusa* [41], to C-H stretching vibrations at 2900 cm^−1^ in pullulan [42], and to C=O vibrations at peaks of 1734, 1652, 165, and 1581 cm^−1^ in Qingzhuan brick tea [43]. These results indicated that free carboxyl and uronic acid may be present in the sample [44, 45]. The absorption peaks of AAPS and the AAPS-iron(III) complex at 1413, 1405, 1323, 1253, 1065, and 1024 cm^−1^ were primarily C-O and C-C vibrations, as well as flexural vibrations of C-C-H and C-O-H, indicating the existence of a pyran-ring structure [46]. In our study, evident changes in the shape of the peaks and the signal intensity of AAPS and the AAPS-iron(III) complex were observed in the range of 2000–800 cm^−1^ (Figure 4). These changes are likely due to the complexation of AAPS with iron ions under alkaline conditions. The absorption peaks at 860 and 434 cm^−1^ of the AAPS-iron(III) complex are considered as the characteristic absorption peaks of FeOOH [38]. In our study, weak absorption peak and no clear absorption peak were observed at 860 and 434 cm^−1^, respectively (Figure 4), indicating that the structure of the AAPS-iron(III) complex may be similar to that of FeOOH.

#### 3.2.2. CD Analysis

**Figure 5:**
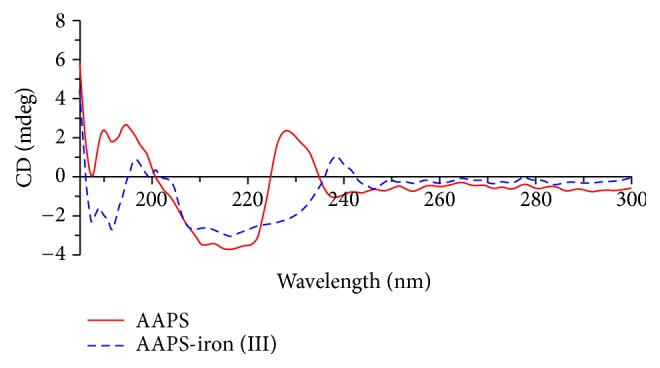
Circular dichroism spectra of AAPS and the AAPS-iron(III) complex.

Circular dichroism spectroscopy is commonly used to detect two dimensional structures of biological macromolecules with optically active groups. Studies have shown that polysaccharides have strong absorption peaks in the far ultraviolet region, especially near 190 nm [47]. Furthermore, the polysaccharides exhibit folding, inversion, entanglement, and irregular morphology due to the interactions between molecules in aqueous solutions. Therefore, the molecular asymmetry occurs, leading to the Cotton effect, and the configuration of asymmetric sugars in solution can be analyzed by circular dichroism spectroscopy [48]. Our results of the circular dichroism chromatograms of AAPS and the AAPS-iron(III) complex showed (Figure 5) that, when the polysaccharide complexed with iron ions, the signal peaks at 190 and 196 nm shifted to 197 and 201 nm, respectively, and the positive peak at 229 nm appeared at 239 nm with reduced signal intensity. It has been reported that metal ions react with carboxyl groups on sugars, resulting in the mutual polymerization of polysaccharide chains to form an “egg box” structure, which leads to increased molecular asymmetry and changes in molecular conformation [49]. Similar results have also been reported by Zhang et al. [50]. Significant shift was not detected between the positive and negative CD spectra of the polysaccharide and its complex with chromium(III) in* Momordica charantia*, indicating that the 3-dimensional spiral structure of the polysaccharide was not changed by the complexing with chromium(III). However, the structural asymmetry of the polysaccharide was enhanced by the formation of the polysaccharide-chromium(III) complex. Furthermore, our study shows that new negative peaks appeared at 187 and 192 nm (Figure 5), indicating that the conformation of the* Auricularia auricula* polysaccharides changed upon the complexation with iron ions. This is probably because the iron(III) ions are bound to the binding sites in the AAPS to form spatially separated iron centers on the polysaccharide backbone.

#### 3.2.3. ^1^H NMR Analysis

**Figure 6:**
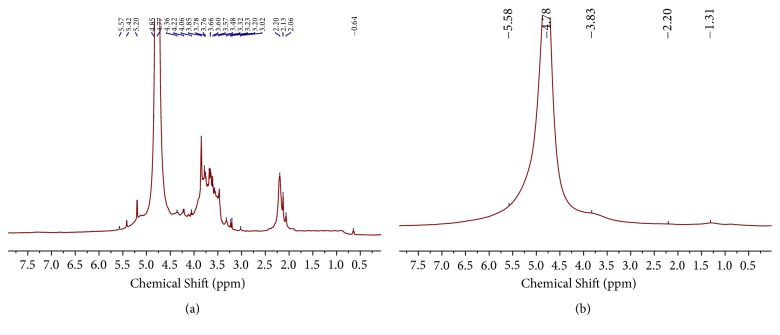
^1^H NMR spectra of AAPS (a) and AAPS-iron(III) complex (b).

In general, the absorption peaks of polysaccharides are mainly distributed in the region of *δ* 4.0-5.5 ppm in the ^1^H NMR spectrum, and more specifically, H-1 is primarily distributed in the region of *δ* 4.8-5.5 ppm, and H-2 and H-6 in the region of *δ* 4.0-4.8 ppm [51]. In our study, the ^1^H NMR spectrum of AAPS (Figure 6(a)) showed that the absorption peaks at *δ* 5.57, 5.42, 5.20, and 4.85 ppm are in the region of heterotopic protons, indicating that there are at least four sugar residues in AAPS. Based on the previous studies [52], it can be predicted from the chemical shift of the protons that AAPS has an alpha glycosidic bond. Furthermore, the hydrogens on C-2 to C-6 of glucose are found at *δ* 3.78-3.12 ppm, the resonance peak at *δ* 3.36 ppm belongs to the signal of H-2 on the glucuronic acid residue, and the resonance peak at *δ* 0.64 ppm presumably represents the signal of a methyl hydrogen. In contrast to AAPS, the ^1^H NMR spectrum of the AAPS-iron(III) complex (Figure 6(b)) showed no clear resonance absorption peaks except for the residual peak of H_2_O near *δ* 4.78 ppm. Similar observations have also been reported previously by Bertini et al. [53] and Wang et al. [38]. This ^1^H NMR invisibility in AAPS-iron(III) complex could be because the ferrous metal is paramagnetic and interferes with the magnetic environment around the ligand, consequently, affecting other peaks on the ligand, lengthening the relaxation time, and broadening the spectral peaks. The NMR signals of the nonmetal bonded sugars were broadened and downfield-shifted (Figure 6(b)), an observation known as the Evans effect [54]. In our study, the presence of the “blind zone,” i.e., the disappearance of absorption peaks caused by the complexation between AAPS and iron(III) (Figure 6(b)), suggests that the AAPS is bonded to iron(III) in the AAPS-iron(III) complex. These results further indicate that AAPS could be potentially used as an effective chelator of iron(III) for a novel human oral iron supplement.

### 3.3. Iron Release In Vitro Assay of the AAPS-Iron(III) Complex

**Figure 7:**
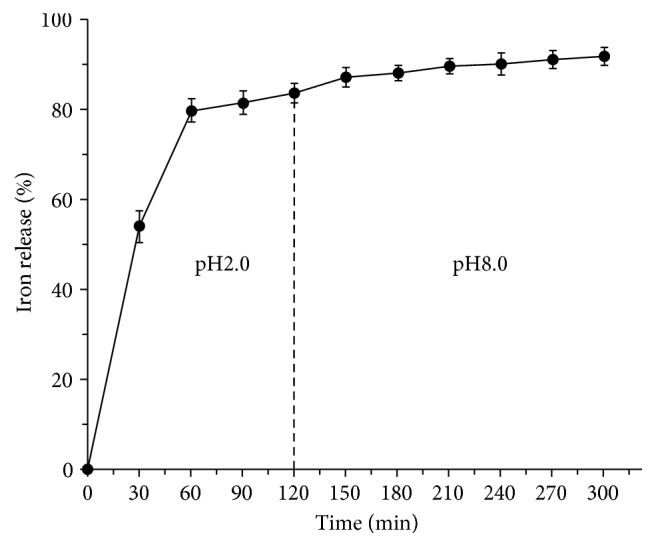
Iron release from the AAPS-iron(III) complex in artificial gastric juice (pH 2.0) and artificial intestinal juice (pH 8.0).

The dissolution of the iron ions in the AAPS-iron(III) complex was investigated in an environment with a similar pH value to that found in human gastrointestinal digestion (Figure 7). Results showed that 53.9% of the iron ions were released in 30 min after the AAPS-iron(III) complex was dissolved in simulated gastric juice. A total of 76.7% and 83.1% of the iron ions were released in 60 min and 90 min, respectively. The relative release of iron ions in the AAPS-iron(III) complex was nearly complete in 120 min with 86.7% of the iron ions released. Upon transferring to an artificial intestinal juice, the amount of iron released from the AAPS-iron(III) complex increased to 93.1% in 3 h, indicating that the AAPS-iron(III) complex exhibits higher water solubility in the environment of a gastrointestinal tract and therefore showing higher bioavailability, which is beneficial for increasing iron absorption. The high bioavailability was also reported in previous studies using in vitro simulating digestive experiments [17, 37] where comparable amount of iron of 87.42% and 91.74% released in 5 h in the AAPS-iron(III) complexes in* Grifola frondosa* and* Astragalus membranaceus*, respectively. These results demonstrate that the AAPS-iron(III) complex could be potentially an efficient candidate for iron supplementation in the treatment of iron deficiency anemia.

### 3.4. Antioxidant Activities of the AAPS-Iron(III) Complex

#### 3.4.1. DPPH Radical Scavenging Activity

**Figure 8:**
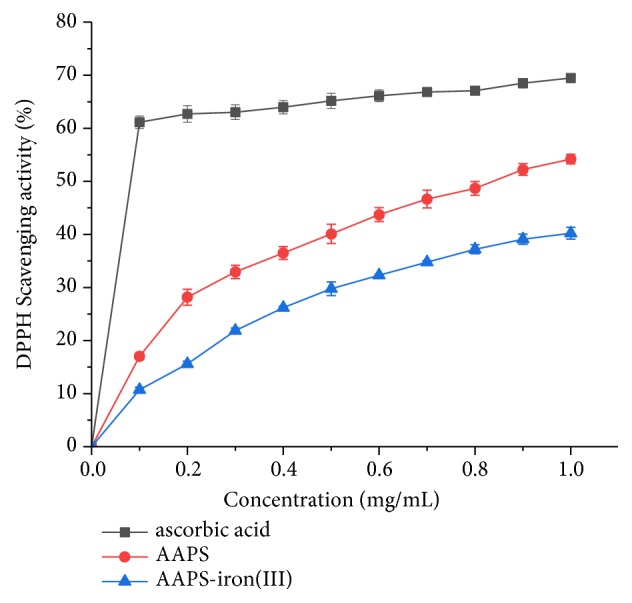
DPPH radical scavenging activity of AAPS, AAPS-iron(III) complex, and ascorbic acid.

**Figure 9:**
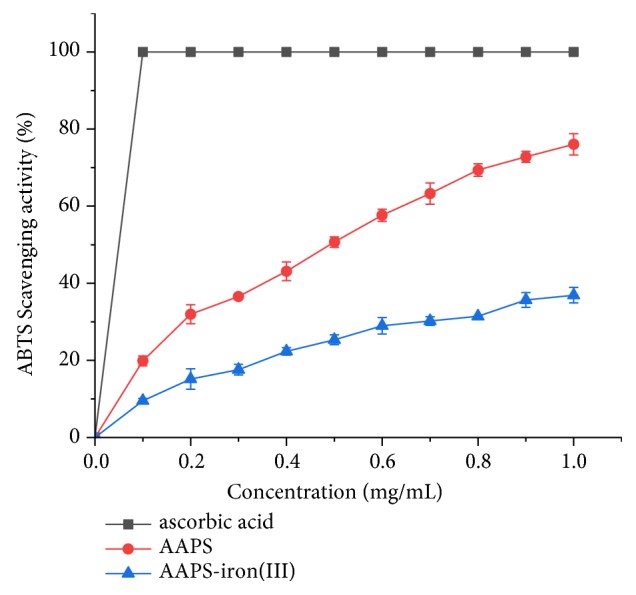
ABTS radical scavenging activity of AAPS, AAPS-iron(III) complex, and ascorbic acid.

**Figure 10:**
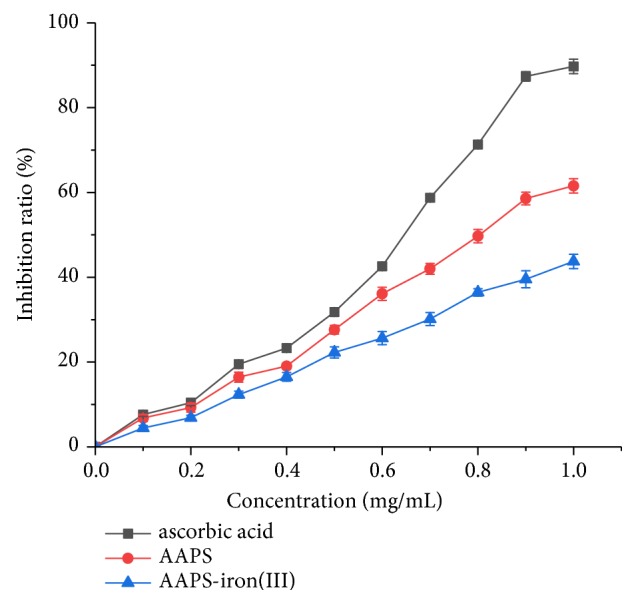
Lipid peroxidation inhibition activity of AAPS, AAPS-iron(III) complex, and ascorbic acid.

The DPPH radical scavenging activity is generally used as one of the indicators to evaluate the antioxidant activity of biochemical compounds. When DPPH radicals react with antioxidants, its color changes from dark purple to bright yellow. The DPPH radical scavenging activity of the substance can be determined by measuring the absorbance at 517 nm. Decreased absorbance at 517 nm indicates strong antioxidant capacity in the sample and high DPPH radical scavenging activity [55]. In our study, both AAPS and the AAPS-iron(III) complex showed strong and concentration-dependent DPPH radical scavenging activities (Figure 8). As the concentrations of the compounds increase, the corresponding DPPH radical scavenging rates increase gradually, showing the solubility dependence at a given concentration. At 1.0 mg/mL (the highest concentration of the samples tested in our study), the DPPH radical scavenging activities of AAPS and the AAPS-iron(III) complex reached their highest rates of 53.8% and 39.5%, respectively. The EC50 values of ascorbic acid, AAPS, and AAPS-iron(III) complex were 0.057 mg/mL, 0.190 mg/mL, and 0.272 mg/mL, respectively. In our study, the polysaccharide solution showed high viscosity with the concentration of the polysaccharide solution higher than 1 mg/mL, significantly affecting the results of the in vitro antioxidant experiments by causing unpredictable readings of the measurements. Therefore, the highest concentration was set up at 1 mg/mL as reported in the previous studies [26, 56]. Furthermore, it was observed from the antioxidant experiments in our study that the AAPS-iron(III) complex clearly obtained the antioxidant capability of the polysaccharide with evident trend of concentration dependence at 1 mg/mL (Figures 8–10), even though the trend of antioxidant results could potentially continue to rise if the concentrations increased. As having antioxidant capacity is often a desirable feature for food and nutritional supplements, these antioxidant activities observed in our study suggest the additional benefits of the AAPS-iron(III) complex over many other typical but nonantioxidant iron supplements, such as the inorganic ferrous sulfate and ferrous fumarate.

#### 3.4.2. ABTS Radical Scavenging Activity

ABTS is shown to react with K_2_S_2_O_8_ to form stable cationic free radicals with an evident absorption peak at 734 nm, while antioxidants effectively inhibit the cationic free radicals, resulting in the decreased absorbance at 734 nm [33]. In our study, the ABTS radical scavenging activities of AAPS and the AAPS-iron(III) complex exhibit a concentration-dependent trend (Figure 9) similar to that of their DPPH radical scavenging activities (Figure 8). At the concentration of 1.0 mg/mL, the ABTS free radical scavenging activity of AAPS-iron(III) and AAPS reached 35.9% and 74.8%, respectively. The EC50 values of ascorbic acid, AAPS, and AAPS-iron(III) complex were 0.050 mg/mL, 0.322 mg/mL, and 0.318 mg/mL, respectively. Similar results were also reported in a recent study [26], where the antioxidant activities in the* Flammulina velutipes* polysaccharides-iron(III) complex were concentration-dependent and were also lower than those in their polysaccharides. These results show that the AAPS-iron(III) complex demonstrates more health benefits in comparison to the traditional iron supplements with no antioxidant capacity.

#### 3.4.3. Inhibition Assay on Liver Lipid Peroxidation

Iron ions catalyze the production of endogenous free radicals in the body, leading to lipid peroxidation in the cell membranes, subsequently causing cytotoxicity and damage to the biological systems [57]. In general, the increased amount of iron in body provided by the traditional iron supplements damages the biological systems. In our study, the AAPS-iron(III) complex showed a concentration-dependent inhibitory activity on liver lipid peroxidation (Figure 10). At the concentration of 1.0 mg/mL, the inhibition rate of the AAPS-iron(III) complex on liver lipid peroxidation reached its highest at 43.1%. The EC50 values of ascorbic acid, AAPS, and AAPS-iron(III) complex were 0.614 mg/mL, 0.537 mg/mL, and 0.493 mg/mL, respectively. Zhang et al. [58] previously reported that the polysaccharide-zinc inclusion complex in* Dioscorea opposite* could significantly reduce the MDA contents and increase the activities of both the superoxide dismutase (SOD) and the total antioxidant capacity (T-AOC) in rats, demonstrating that the oral administration of polysaccharide-zinc could suppress the oxidant stress in liver of rats. These results demonstrate that, due to its low oxidative toxicity, the AAPS-iron(III) complex could be consumed at higher dosage levels than other traditional supplements such as ferrous sulfate without causing the side effects. With the intake of traditional iron supplements in the treatment of patients with iron deficiency anemia, the patients would show the rapid increase of iron levels in body to cause the lipid oxidative stress, resulting in a wide range of side effects in patients such as diarrhea, nausea, or vomiting. The antioxidant activity of the AAPS-iron(III) complex could inhibit the lipid oxidative stress caused by iron intake in patients and reduce the side effects occurring during the treatment. Furthermore, in comparison to the traditional iron supplements, more iron could be absorbed and the treatment cycle could be shortened in patients treated with AAPS-iron(III) complex. Moreover, the antioxidant activities of the polysaccharide retained by the AAPS-iron(III) complex could also have other positive effects on the patients, such as the enhancement of the antioxidation and immunity. Therefore, the AAPS-iron(III) complex has showed the promising potential to be the novel type of iron supplements in the treatment of patients with iron deficiency anemia.

### 3.5. Antianemia Function Test of the AAPS-Iron(III) Complex

The results of the antianemia tests showed that the AAPS-iron (III) complex demonstrated a high efficiency on iron deficiency anemia (Table 1). For example, the increase of body weight in the MC group of the model rats of iron deficiency anemia is significantly larger than that in BC group (p<0.05), while, in 35 days, the increase of body weight in both HI and PC groups was much larger than that in the MC group (p<0.01). Furthermore, many of the blood parameters in the MC group were significantly lower than those in the BC group (p<0.05). The amount of FP in the MC group was significantly larger than that in the BC group (p<0.01), indicating that the rats in the MC group were iron deficient and in line with the characteristics of iron deficiency anemia. Although many of the values of the blood parameters increased and the amount of FP decreased in the LW group in comparison to the MC group, these variations were not statistically significant. Similarly, many of the values of the blood parameters increased and the amount of FP decreased in the MI group in comparison to the MC group; however, only the variations in FP and HCT were statistically significant (p<0.05). Furthermore, the amount of FP in both the HI and MI groups was significantly lower than that in the MC group (p<0.05). In comparison to the MC group, both the HI and PC groups showed largely increased values in all of the blood parameters examined and statistical significance was identified in the HI group (p<0.05) with similar values to those in the BC group. In the HI group, the body weights of the animals increased evidently, with overall apparent improvements in behaviors, activities, and hair color. These data clearly demonstrate the significant improvement on the various blood parameters in the rats of iron deficiency anemia by the* Auricularia auricula* polysaccharide-iron(III) complex, consequently improving the conditions of iron deficiency. These results are in line with those in a recent study [59], where the* Enteromorpha* polysaccharide-iron(III) complex significantly improved the blood parameters and controlled the body weight in the rats of iron deficiency anemia and further revealed the dosage-dependent relationships with the treatment effect. The APPS-iron(III) complex shows great potential for the development of a safe and an efficient iron supplement.

## 4. Conclusions

In this study, a novel* Auricularia auricula* polysaccharide-iron(III) complex was prepared and characterized, and the synthetic conditions were optimized. The AAPS-iron(III) complex was a reddish brown powder with an iron content of 28.4%, high water solubility, and high thermal stability in the temperature range of 50–256°C. As determined from the spectral data, the iron(III) ions in the AAPS-iron(III) complex were bound to the binding sites in the AAPS to form spatially separated iron centers on the polysaccharide backbone. The complex exhibited high digestion efficiency in vitro as well as high antioxidant activity. Therefore, the AAPS is considered potentially as a ligand for chelating iron to promote digestion and absorption, which could be a great benefit for treatment of iron deficiency anemia. Furthermore, the AAPS-iron(III) complex has the advantages of reduced side effects and oxidative stresses caused by overload of iron in comparison with other currently available iron supplements. Specifically, the* Auricularia auricula* AAPS-iron(III) complex significantly improved the blood parameters and body weights in the model rats of iron deficiency anemia. These results suggest that the AAPS-iron(III) complex can be potentially used as a nutritional fortifier for iron supplement in industrial processing and to assist the treatment of iron deficiency anemia.

## Figures and Tables

**Table 1:** Effects of AAPS-iron(III) complex on blood parameters and body weight in the model rats of iron deficiency anemia. Measurements are given as average ± standard deviation (n=10). Statistical significance is indicated by a (p<0.01) and b (p<0.05) in comparison to the BC group and c (p<0.05) and d (p<0.01) in comparison to the MC group, respectively.

Group	Time (day)	HB (g/L)	RBC (×10^12^)	FP (*μ*g/L)	HCT (%)	MCV (fL)	MCH (pg)	MCHC (g/L)	Weight (g)
MC	0	110.22±5.91^a^	4.48±0.63^a^	73.53±8.68^a^	32.15±1.95^a^	51.30±3.09^b^	14.35±0.54^b^	310.20±5.72^b^	185.25±16.2^b^
	35	108.68±7.23	4.41±0.66	75.21±9.28	29.89±2.60	42.12±2.77	14.16±0.83	306.83±6.94	175.24±5.24
BC	0	125.43±7.67	6.97±0.60	53.07±9.71	42.57±2.86	51.40±1.89	19.04±0.62	325.17±7.32	215.72±17.30
	35	126.04±8.03	7.07±0.72	53.41±9.49	43.04±3.14	50.12±1246	18.85±0.66	325.19±7.36	225.24±16.17
PC	0	110.35±6.83^b^	4.50±0.56^b^	71.87±6.09^a^	33.25±6.11^b^	51.19±3.68^b^	14.04±0.62^b^	311.17±7.32^b^	186.35±12.3^b^
	35	121.02±3.12^c^	6.53±0.72^d^	54.26±4.38^d^	42.52±2.38^c^	49.26±2.68^c^	16.85±0.66^c^	325.19±7.36^c^	220.72±18.55^d^
LW	0	110.83±6.27^b^	4.61±0.57^b^	74.89±7.82^a^	30.44±3.73^b^	50.11±3.47^b^	14.27±0.40^b^	311.86±7.37^b^	187.26±16.26^b^
	35	112.46±7.30	4.65±0.58	70.23±7.92	33.41±3.11	42.30±1.96	14.92±0.55	312.06±8.49	192.02±18.04
MI	0	110.80±7.03^b^	4.43±0.72^a^	72.17±6.95^a^	31.09±1.78^a^	51.46±2.32^b^	14.78±0.39^b^	313.43±6.54^b^	185.92±15.09^b^
	35	115.93±6.55	5.11±0.69	65.99±8.74^c^	39.24±2.38^c^	43.94±2.15	15.04±0.46	317.00±7.77	201.46±17.93
HI	0	109.55±6.52^b^	4.42±0.86^a^	71.44±9.09^a^	32.50±2.74^b^	51.43±2.75^b^	14.20±0.63^b^	309.87±8.42^b^	186.43±15.44^b^
	35	119.63±6.96^c^	6.1290.65^c^	58.86±9.34^c^	41.17±3.55^c^	48.85±3.0^c^	15.49±0.71^c^	322.47±8.08^c^	218.75±18.09^d^

## Data Availability

The data used to support the findings of this study are available from the corresponding author upon request.
